# Functional K_Ca_3.1 K^+^ channels are required for human fibrocyte migration

**DOI:** 10.1016/j.jaci.2011.07.047

**Published:** 2011-12

**Authors:** Glenn Cruse, Shailendra R. Singh, S. Mark Duffy, Camille Doe, Ruth Saunders, Chris E. Brightling, Peter Bradding

**Affiliations:** Department of Infection, Immunity and Inflammation, Institute for Lung Health, University of Leicester, Leicester, United Kingdom

**Keywords:** Pulmonary fibrosis, asthma, fibrocyte, cell migration, ion channel, K_Ca_3.1, patch clamp electrophysiology, 1-EBIO, 1-Ethyl-2-benzimidazolinone, αSMA, α-Smooth muscle actin, ASM, Airway smooth muscle, IPF, Idiopathic pulmonary fibrosis, *K*_d_, Concentration producing 50% block

## Abstract

**Background:**

Fibrocytes are bone marrow–derived CD34^+^ collagen I–positive cells present in peripheral blood that develop α-smooth muscle actin expression and contractile activity in tissue culture. They are implicated in the pathogenesis of tissue remodeling and fibrosis in both patients with asthma and those with idiopathic pulmonary fibrosis. Targeting fibrocyte migration might therefore offer a new approach for the treatment of these diseases. Ion channels play key roles in cell function, but the ion-channel repertoire of human fibrocytes is unknown.

**Objective:**

We sought to examine whether human fibrocytes express the K_Ca_3.1 K^+^ channel and to determine its role in cell differentiation, survival, and migration.

**Methods:**

Fibrocytes were cultured from the peripheral blood of healthy subjects and patients with asthma. Whole-cell patch-clamp electrophysiology was used for the measurement of ion currents, whereas mRNA and protein were examined to confirm channel expression. Fibrocyte migration and proliferation assays were performed in the presence of K_Ca_3.1 ion-channel blockers.

**Results:**

Human fibrocytes cultured from the peripheral blood of both healthy control subjects and asthmatic patients expressed robust K_Ca_3.1 ion currents together with K_Ca_3.1 mRNA and protein. Two specific and distinct K_Ca_3.1 blockers (TRAM-34 and ICA-17043) markedly inhibited fibrocyte migration in transwell migration assays. Channel blockers had no effect on fibrocyte growth, apoptosis, or differentiation in cell culture.

**Conclusions:**

The K^+^ channel K_Ca_3.1 plays a key role in human fibrocyte migration. Currently available K_Ca_3.1-channel blockers might therefore attenuate tissue fibrosis and remodeling in patients with diseases such as idiopathic pulmonary fibrosis and asthma through the inhibition of fibrocyte recruitment.

Fibrocytes are bone marrow–derived CD34^+^ collagen I–positive cells present in peripheral blood that develop α-smooth muscle actin (αSMA) expression and contractile activity in tissue culture. Their recruitment to sites of wound repair and fibrosis occurs in many diseases and experimental models,^1^ including murine radiation- and bleomycin-induced lung fibrosis.^2,3^ Fibrocytes are present in human idiopathic pulmonary fibrosis (IPF) tissue,^4^ and patients with IPF have 10-fold more fibrocytes in their peripheral circulation than healthy control subjects.^5^ Human fibrocytes were recruited to the lungs of mice with severe combined immunodeficiency in response to bleomycin,^6^ and fibrocytes are present in the airways of asthmatic patients.^7^ The number of peripheral blood fibrocytes is increased in asthmatic patients^7^ and related to the degree of airflow obstruction.^8^ In addition, we have identified CD34^+^ αSMA^+^ cells within the airway smooth muscle (ASM) bundles in asthmatic patients.^9^ Inhibiting fibrocyte recruitment and migration therefore has great potential for the treatment of many diverse diseases characterized by tissue fibrosis and remodeling.

The mechanisms controlling fibrocyte migration to tissue are poorly defined. Fibrocytes express CCR7, CXCR3, CXCR4, CCR5, and CCR3,^6,9,10^ suggesting they have the potential to migrate toward a number of chemokines. They also migrate in response to the complex milieu of ASM-conditioned medium, an effect mediated in part by platelet-derived growth factor.^9^ Thus although inhibiting individual chemoattractants might attenuate fibrocyte migration under specific circumstances, redundancy *in vivo* might weaken such an approach.

Ion channels are emerging as interesting therapeutic targets in both inflammatory and structural nonexcitable cells. Channels carrying K^+^, Cl^−^, and Ca^2+^ mediate a variety of cell processes, including proliferation,^11^ differentiation,^12^ adhesion,^13^ mediator release,^14^ and migration.^15^ The ion-channel repertoire of human fibrocytes is unknown. In this study we demonstrate for the first time that human fibrocytes express the Ca^2+^-activated K^+^ channel K_Ca_3.1 and that K_Ca_3.1 blockade markedly attenuates fibrocyte migration in response to the complex milieu of human ASM-conditioned medium and CXCL12.

## Methods

Full experimental details are provided in the Methods section in this article’s Online Repository at www.jacionline.org.

### Subjects

Asthma was defined as described previously.^16^ Healthy subjects had no history of respiratory disease. Participants were nonsmokers and free from exacerbations for at least 6 weeks. The Leicestershire Research Ethics Committee approved the study (reference no. 4977). All subjects provided written informed consent.

### Fibrocyte isolation and culture

Fibrocytes were isolated from peripheral blood and cultured as described previously.^9^ Fibrocyte purity and differentiation were assessed by means of flow cytometry and immunofluorescent staining for CD34, αSMA, and collagen I, as described previously.^9^

### Quantitative real-time RT-PCR

Quantitative real-time reverse transcription PCR for K_Ca_3.1 was performed as previously described.^17^

### K_Ca_3.1 protein expression

K_Ca_3.1 protein expression was analyzed by means of Western blotting with validated rabbit anti-human K_Ca_3.1 antibodies (M20; Dr M. Chen, GlaxoSmithKline, Stevenage, United Kingdom)^18^ and P4997 (Sigma-Aldrich, Poole, United Kingdom). K_Ca_3.1 expression was also examined by means of immunofluorescence with the same M20 and P4997 antibodies; the methods are as previously described.^19^

### Patch-clamp electrophysiology

The whole-cell variant of the patch-clamp technique was used, as previously described.^14^

### Fibrocyte migration

First, we used a 24-well transwell migration assay to measure the migration of detached differentiated fibrocytes that had been in culture for 1 week after isolation. Cells were placed in the top of the transwell, and for the chemoattractant in the lower chamber, we used conditioned medium from TNF-α–activated ASM cultures. Chemokines present in this media include the fibrocyte chemoattractant CXCL12.^20^ Cells were incubated in the presence of chemoattractant for 4 hours, at which point cells that had migrated into the lower well were counted by a blinded observer. Where required, the specific K_Ca_3.1 blockers TRAM-34 (Professor H. Wulff, University of California, Davis, Calif)^21^ and ICA-17043 (Senicapoc; Icagen, Inc, Durham, NC)^22^ were added to the top chamber before migration. These drugs did not act as chemoattractants when added to media in the bottom chamber.

In a further set of migration experiments, freshly isolated PBMCs containing the immature fibrocyte population were allowed to migrate through a transwell, as described above, with the established fibrocyte chemoattractant CXCL12 placed in the lower chamber.^6^ Where required, the K_Ca_3.1 blocker TRAM-34 was added to the top chamber before migration. After 4 hours, the transwell was discarded, and the migrated cells were cultured for a further 7 days. The adherent cells were then fixed in 4% paraformaldehyde, stained for collagen I, and then counted by a blinded observer.

A second migration assay was used to investigate the migration of adherent elongated fibrocytes, as described previously.^9^

### Fibrocyte growth and maturation

Fibrocytes were incubated with TRAM-34 (20-2000 nmol/L) either from days 0 to 7 or from days 7 to 14 after isolation. Cells were then harvested and counted. In addition, the MTS proliferation assay was performed. Parallel chamber slides were examined to look at fibrocyte morphology and differentiation markers, as previously described.^9^

### Quantification of cell death

Fibrocytes were incubated with TRAM-34 at a dose range of 20 to 2000 nmol/L in their normal growth medium, either from days 0 to 7 after isolation or from days 7 to 14. Cells were analyzed by means of flow cytometry for the presence of early apoptotic and late apoptotic cells.

### Statistics

Experiments were performed in duplicate, and a mean value was derived for each condition. For all assays, across-group differences were analyzed by means of ANOVA, and inhibition of migration was analyzed by using a paired *t* test. Patch-clamp data were analyzed by using paired or unpaired *t* tests, as appropriate.

## Results

### Human fibrocytes express K_Ca_3.1 mRNA and protein

**Fig 1:**
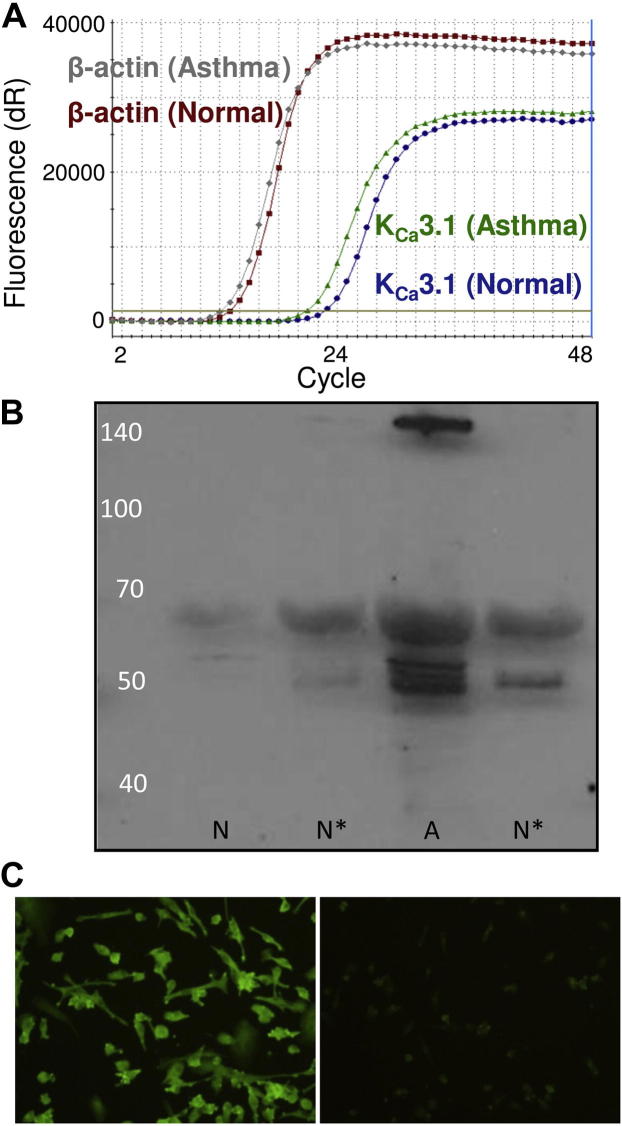
K_Ca_3.1 mRNA and protein expression in fibrocytes. **A,** Real-time RT-PCR demonstrating K_Ca_3.1 mRNA expression in fibrocytes from 1 healthy and 1 asthmatic donor. Data are representative of results from 9 donors. **B,** Western blotting for K_Ca_3.1 (P4997 antibody) in healthy *(N)* and asthmatic *(A)* fibrocytes demonstrating bands of approximately 53 kd. ∗Same donor. **C,** Immunofluorescent staining (M20 antibody) for K_Ca_3.1 in cultured fibrocytes *(left panel)* demonstrating strong immunoreactivity. Results of isotype control staining were negative *(right panel)*.

Fibrocyte cell cultures developed the typical morphology and immunologic markers of human fibrocytes.^9^ All fibrocyte cultures assessed by quantitative real-time reverse transcription PCR (n = 6 healthy subjects, n = 3 asthmatic patients) expressed K_Ca_3.1 mRNA (Fig 1, *A*). However, there was no difference in K_Ca_3.1 mRNA expression between healthy subjects and asthmatic patients by using the mean K_Ca_3.1 cycle threshold/β-actin cycle threshold values (mean ± SEM: healthy subjects, 0.604 ± 0.023 [n = 6]; asthmatic patients, 0.606 ± 0.022 [n = 3]). Fibrocyte lysates contained a K_Ca_3.1 immunoreactive protein of approximately 53 kd in molecular weight (Fig 1, *B*), which is close to the predicted size of 47 kd and similar to the previously reported size of 53 kd for K_Ca_3.1 in Western blots.^17,18^ This was identified by both the M20 K_Ca_3.1 antibody, which targets the N terminus (n = 9 subjects), and the P4997 antibody, which targets the C terminus (n = 3 subjects). P4997 also produced a band of approximately 65 kd of uncertain significance. K_Ca_3.1 immunoreactivity was also evident in fixed cultured fibrocytes by using both K_Ca_3.1 antibodies (Fig 1, *C*).

### Human fibrocytes express heterogeneous ion currents at “rest”

**Fig 2:**
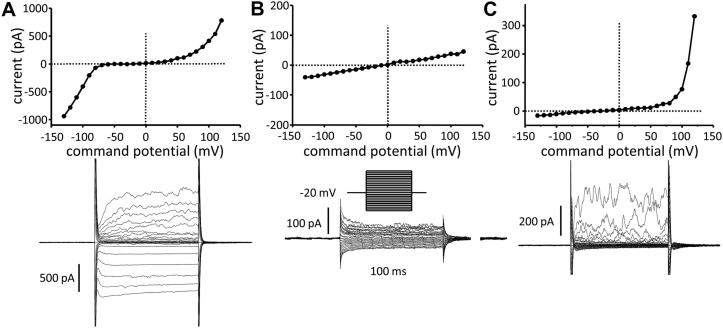
Heterogeneity of resting whole-cell currents in human fibrocytes. Example current-voltage (I/V) curves *(top panels)* and raw current *(bottom panels)* of an inwardly rectifying current **(A)**, a linear current **(B)**, and an outwardly rectifying current **(C)** are shown.

At baseline, immediately after obtaining the whole-cell patch-clamp configuration, unstimulated fibrocytes (n = 30 healthy cells from 7 donors and n = 17 asthmatic cells from 4 donors) demonstrated heterogeneity of the resting whole-cell current, with a small outwardly rectifying current in 91% of cells, a steeply inwardly rectifying current characteristic of the Kir2.0 family of channels in 30% of cells, and a linear current in 9% of cells (Fig 2). The outwardly rectifying current was not altered by substitution of external Cl^−^ (data not shown) and might be carried by a nonselective cation channel, such as the ubiquitous transient receptor potential channel 7.^23^ Mean reversal potential for the whole population of cells (n = 47) at baseline was −29.8 ± 3.0 mV. Mean baseline whole-cell current at +40 mV was 122 ± 25 pA, and there was no difference in the size of the baseline current or reversal potential in cells from healthy compared with asthmatic donors. There was no evidence in any cell for a current carried by the voltage-dependent K^+^ channel K_v_1.3, which is found in resting T cells.^24^

Measurements of fibrocyte capacitance were unreliable because of the relatively large cell size. However, cell size assessed by means of flow cytometry was similar between donors (mean ± SEM: 4891 ± 158 arbitrary units [n = 13 donors]) and between healthy control subjects compared with asthmatic patients (*P* = .60).

### Human fibrocytes express robust K_Ca_3.1 ion currents

To elicit K_Ca_3.1 currents, we used the K_Ca_3.1 opener 1-ethyl-2-benzimidazolinone (1-EBIO; Tocris, Avonmouth, United Kingdom), as described previously.^17,25,26^ This compound opens K_Ca_3.1 with a half-maximal value of about 30 μmol/L for heterologously expressed K_Ca_3.1.^27^

**Fig 3:**
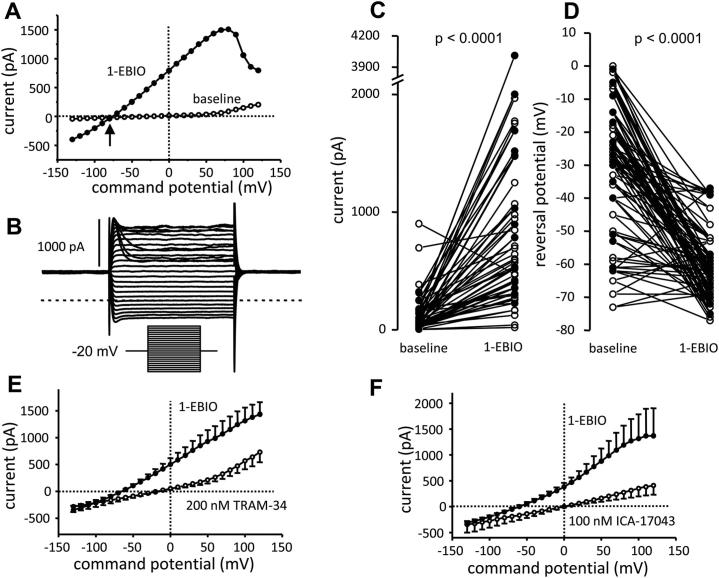
Human fibrocytes express robust K_Ca_3.1 currents. **A,** Current-voltage (I/V) curve of a fibrocyte at baseline and after addition of the K_Ca_3.1 opener 1-EBIO (100 μmol/L). 1-EBIO induces a large whole-cell current characteristic of K_Ca_3.1, with an accompanying negative shift in reversal potential *(arrow)*. **B,** Characteristic K_Ca_3.1 raw current from the same cell as in Fig 3, *A*, after 1-EBIO. The *dotted line* represents zero current at reversal potential. *Inset*, Voltage protocol. **C**, Whole-cell current measured at +40 mV in fibrocytes from healthy *(open circles)* and asthmatic *(solid circles)* subjects at baseline and after addition of 1-EBIO (100 μmol/L). **D,** Reversal potential in fibrocytes from healthy *(open circles)* and asthmatic *(solid circles)* subjects at baseline and after addition of 1-EBIO. **E,** Block of K_Ca_3.1 whole-cell currents by TRAM-34 (200 nmol/L) with an accompanying positive shift in reversal potential *(arrow)*. Mean ± SEM of 18 cells recorded. **F,** Block of K_Ca_3.1 whole-cell currents by ICA-17043 (100 nmol/L) with an accompanying positive shift in reversal potential *(arrow)*. Mean ± SEM of 8 cells recorded.

Addition of 1-EBIO (100 μmol/L) elicited a typical K_Ca_3.1 current in 46 of 47 cells tested (from 7 healthy and 4 asthmatic donors; Fig 3, *A-C*). Whole-cell current at +40 mV increased from a mean ± SEM of 122 ± 25 pA before 1-EBIO to 795 ± 104 pA after 1-EBIO (*P* < .0001; Fig 3, *C*) accompanied by a negative shift in reversal potential from −29.8 ± 3.0 mV to −61.8 ± 1.6 mV (*P* < .0001; Fig 3, *D*). 1-EBIO induced a greater current in fibrocytes from asthmatic patients (1-EBIO–dependent current at +40 mV = 969 ± 216 pA [n = 17 cells]) compared with that seen in fibrocytes from healthy subjects (1-EBIO–induced current = 506 ± 94 pA [n = 30 cells], *P* = .029), but there was no difference in reversal potential (*P* = .352).

The 1-EBIO–induced current demonstrated the classical electrophysiological features of K_Ca_3.1: it appeared immediately as voltage steps were applied, did not decay during a 100-ms pulse, and demonstrated inward rectification from membrane potentials positive to about +40 mV (Fig 3, *A* and *B*). Furthermore, the current was completely blocked by the selective K_Ca_3.1 blockers TRAM-34 (concentration producing 50% block [*K*_d_], 20 nmol/L)^21^ and ICA-17043 (*K*_d_, approximately 10 nmol/L)^22^ at concentrations of 200 and 100 nmol/L, respectively (Fig 3, *E* and *F*). See the Results section in this article’s Online Repository at www.jacionline.org for statistical analysis. The reversal potentials after addition of TRAM-34 and ICA-17043 (in the presence of 1-EBIO) were significantly more positive (*P* = .0018 and *P* = .0016, respectively) than the respective baseline values, indicating the presence of open K_Ca_3.1 channels at rest. In summary, human fibrocytes express robust K_Ca_3.1 currents.

### K_Ca_3.1 channels do not regulate fibrocyte growth, maturation, or survival

**Fig 4:**
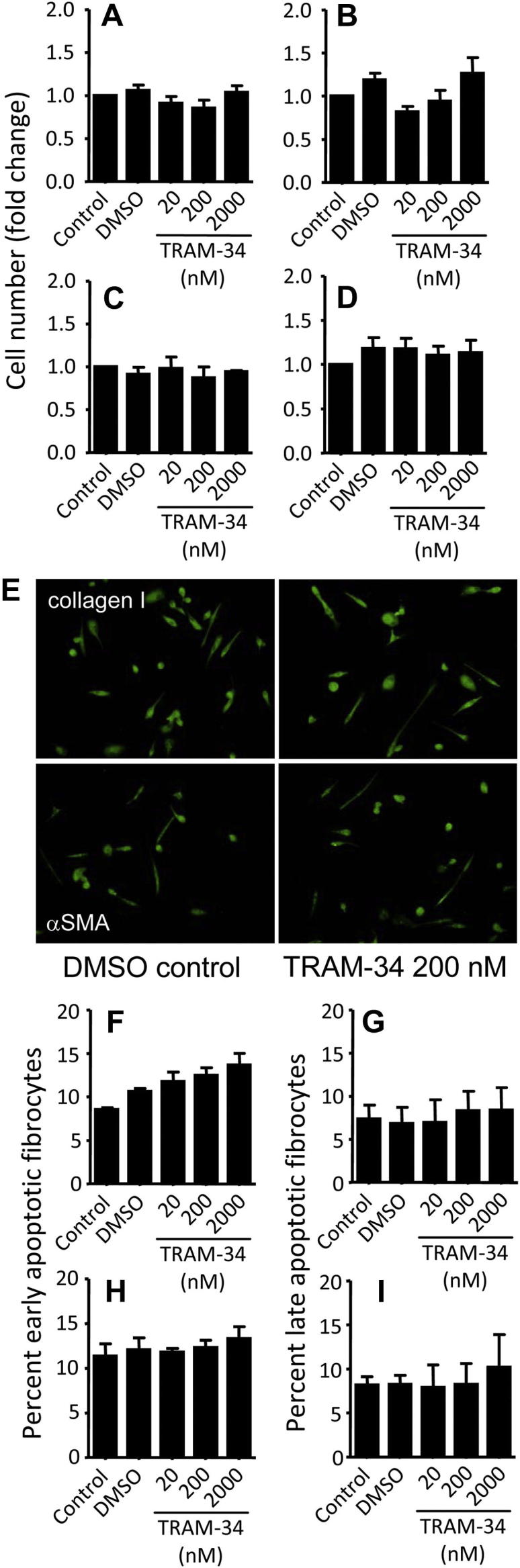
K_Ca_3.1 blockade does not affect fibrocyte growth, differentiation, or survival. **A** and **B,** Fold increase (over control media) in the number of fibrocytes incubated with dimethyl sulfoxide *(DMSO)* control and TRAM-34 (20-2000 nmol/L) from days 0 to 7 (Fig 4, *A*) and from days 7 to 14 (Fig 4, *B*) after isolation (n = 7 donors). Values are expressed as means ± SEMs. **C** and **D,** Cell proliferation and viability assessed by using the MTS assay in fibrocytes incubated with DMSO control and TRAM-34 (20-2000 nmol/L) from days 0 to 7 (Fig 4, *C*) and from days 7 to 14 (Fig 4, *D*). Data are presented as the mean fold increase over the media control ± SEM (n = 3). **E,** Fibrocyte morphology after 7 days in control conditions *(left panels)* or with TRAM-34 (200 nmol/L, *right panels*) is similar. Cells in both conditions express the typical spindle-like morphology and express the fibrocyte markers collagen I *(top panels)* and αSMA (*lower panels*, representative of 2 independent experiments). **F-I,** Apoptosis was assessed by means of flow cytometry with fluorescein isothiocyanate–Annexin V/propidium iodide binding in fibrocytes incubated with control media, DMSO control, and TRAM-34 (20-2000 nmol/L) from days 0 to 7 and from days 7 to 14. Percentage of early apoptotic fibrocytes revealed by Annexin V^+^ (Fig 4, *F*: days 0-7; Fig 4, *H*: days 7-14) and late apoptotic cells revealed by Annexin V^+^/propidium iodide–positive cells (Fig 4, *G*: days 0-7; Fig 4, *I*: days 7-14) of 3 fibrocytes incubated with relevant conditions. Data are presented as means ± SEMs.

Incubation of developing fibrocyte cultures with TRAM-34 (20-2000 nmol/L) at days 0 to 7 or days 7 to 14 after isolation had no significant effect on cell number (Fig 4, *A* and *B*), proliferation (Fig 4, *C* and *D*), cell morphology, expression of fibrocyte markers (Fig 4, *E*), or early and late apoptosis (Fig 4, *F-I*).

### Functional K_Ca_3.1 channels are required for human fibrocyte migration

**Fig 5:**
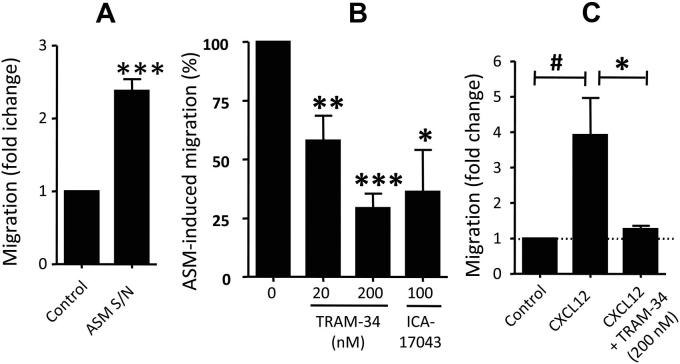
K_Ca_3.1 blockade inhibits human fibrocyte migration. **A**, Migration of cultured, 1-week-old detached fibrocytes through an 8-μm pore-size transwell membrane was significantly enhanced when conditioned media from TNF-α–stimulated ASM *(ASM S/N)* was added to the bottom chamber. Values are presented as means ± SEMs from 9 donors. ∗∗∗*P* < .0001, paired *t* test. **B**, Fibrocyte migration was significantly attenuated by the K_Ca_3.1 blockers TRAM-34 and ICA-17043. ASM-dependent migration in Fig 5, *A*, is represented as 100% in Fig 5, *B*. Dimethyl sulfoxide *(DMSO)* 0.1% was present in all conditions. Values are means ± SEMs from 9 donors. *P* < .0001 across groups, ANOVA. ∗*P* < .05, ∗∗*P* < .01, and ∗∗∗*P* < .0001, paired *t* test. **C**, Migration of freshly isolated fibrocytes/precursors in a PBMC preparation in response to CXCL12 is markedly attenuated by TRAM-34 (200 nmol/L). After 4 hours of migration, the cells were cultured for 1 week, stained for collagen I, and then counted. Values are mean ± SEMs from 7 donors. *P* = .0053 across conditions, ANOVA. #*P* = .003. ∗*P* < .0001, paired *t* test.

ASM supernatants stimulated with TNF-α induced the migration of differentiated 1-week-old fibrocytes (Fig 5, *A*), with migration 2.4 ± 0.2–fold greater than control medium (n = 9 donors, *P* < .0001). The K_Ca_3.1-specific blocker TRAM-34 markedly inhibited the ASM-induced fibrocyte migration dose dependently (Fig 5, *B*). Thus with 200 nmol/L TRAM-34 (*K*_d_, 20 nmol/L),^21^ ASM-induced migration was reduced by 70.7% ± 6.2% (n = 9, *P* < .0001). ASM-induced fibrocyte migration was also inhibited by the distinct and specific K_Ca_3.1 blocker ICA-17043 (*K*_d_, approximately 10 nmol/L),^22^ with migration reduced by 63.8% ± 17.8% at a concentration of 100 nmol/L (n = 5, *P* = .023). Freshly isolated fibrocytes/precursors within a PBMC population migrated in response to CXCL12 (fold change, 3.9 ± 1.0), and this was markedly inhibited by 200 nmol/L TRAM-34 (82.9% ± 4.4% inhibition, *P* = .0001 across groups; Fig 5, *C*).

Migration of adherent elongated fibrocytes was a mean of 25.1 ± 0.9 μm from the point of origin over 4.5 hours. This was not inhibited by TRAM-34 or ICA-17043 (n = 7 donors and n = 3 donors, respectively).

## Discussion

This is the first study to examine ion channel expression in human fibrocytes. The key finding is that human fibrocytes express the calcium-activated K^+^ channel K_Ca_3.1 and that blockade of this inhibits migration of detached motile fibrocytes induced by the complex milieu of TNF-α–activated ASM supernatant and the chemokine CXCL12.

Expression of both K_Ca_3.1 mRNA and protein was evident in fibrocytes, and importantly, functional channels revealed by means of patch-clamp electrophysiology were present in the plasma membrane. Large K_Ca_3.1 currents were present in 98% of cells tested, and this distinguishes fibrocytes from related ASM cells, which only express consistent currents after incubation with growth factors.^17^ K_Ca_3.1 whole-cell currents were larger in cells from asthmatic patients compared with healthy control subjects, although mRNA and protein expression were similar. The biological significance of this requires further investigation.

K_Ca_3.1 activity has been shown to be important for the migration of several cell types, including mast cells,^15^ glial cells,^28^ NIH3T3 fibroblasts,^29^ and melanoma cells.^29^ The proposed mechanism is that K_Ca_3.1 activity is required for detachment of the uropod through the regulation of cell volume and actin dynamics.^30^ In keeping with this, K_Ca_3.1 blockade with the 2 distinct and specific channel blockers TRAM-34 and ICA-17043 resulted in a marked inhibition of fibrocyte migration in transwell migration assays. This was evident when fibrocyte migration was analyzed in both freshly isolated cells within a PBMC preparation and differentiated cells that had been cultured for 1 week. Importantly, the ion-channel blockers inhibited migration at physiologically relevant concentrations. Thus it takes 5 to 10 times the *K*_d_ to inhibit all channels. The *K*_d_ for TRAM-34 is 20 nmol/L,^21^ and that for ICA-17043 is 6 to 10 nmol/L.^22^ At 10 times the *K*_d_ for both blockers, migration was markedly inhibited, and at these concentrations, both drugs are highly specific for K_Ca_3.1.^22,31^

Interestingly, K_Ca_3.1 blockade only inhibited fibrocyte migration when the cells were freshly isolated or had been detached and were thus “rounded” in shape, although not when the cells were adherent and elongated. This has several potential explanations.

First, migration of adherent fibrocytes is relatively poor compared with that of other cells, such as eosinophils, in 2-dimensional and 3-dimensional assays.^9,32^ Thus the propensity for fibrocytes to migrate might be greater when “detached,” thus mimicking circulating cells. This makes sense biologically because peripheral blood fibrocytes need to move through the endothelium to their tissue destination and might only develop their adherent morphology once they reach their goal. In keeping with this, fibrocytes present in human bronchial mucosa 24 hours after allergen challenge have a rounded morphology.^33^ In contrast, fibrocytes present in the airway mucosa from patients with chronic asthma and murine airway mucosa 6 weeks after antigen challenge have the adherent morphology.^9,33^

Second, the signaling pathways for migration might be different in detached fibrocytes compared with those in adherent elongated cells. This is supported by the finding that migration of both freshly isolated and cultured detached fibrocytes is inhibited by K_Ca_3.1 blockers but that of adherent cells is not.

T cells also express K_Ca_3.1 and were present as a contaminant in our fibrocyte cultures. However, K_Ca_3.1 expression in quiescent T cells is relatively low at approximately 20 channels per cell.^24^ Thus although T cells might potentially contribute to the PCR signal we demonstrated, they would not account for the positive K_Ca_3.1 staining in Western blotting and immunofluorescent microscopy. Fibrocytes and T cells were readily distinguishable by size, which is validated by the fact that we never recorded a characteristic T-cell K_v_1.3 current when patch clamping. Therefore although occasional T cells were evident in the migration assays performed on 1-week-old cultured fibrocytes, these were readily apparent and did not complicate the analysis. Thus we are confident that the functional effects we attribute to K_Ca_3.1 in human fibrocytes are not confounded by the presence of contaminating T cells.

Increasing evidence indicates that fibrocytes play an important role in tissue remodeling and fibrosis in both the lung^2-9^ and a variety of other organs.^1,6,34^ Strategies that inhibit fibrocyte migration might therefore attenuate fibrotic disease. Several chemokines have been identified as fibrocyte chemoattractants, but targeting these might be ineffective because of extensive redundancy.^35^ A less selective approach might therefore be more effective. K_Ca_3.1 blockade inhibits cell migration to a number of diverse stimuli^15^ and inhibited fibrocyte migration to the complex milieu of activated ASM supernatant, as well as CXCL12. This suggests that K_Ca_3.1 blockade might be particularly effective at inhibiting fibrocyte migration *in vivo*.

K_Ca_3.1 is an attractive pharmacologic target because its blockade does not have major adverse effects on normal physiology. K_Ca_3.1 knockout mice are viable and of normal appearance, produce normal litter sizes, and exhibit rather mild phenotypes.^36-38^ High doses of TRAM-34 administered to mice over many weeks are well tolerated,^39^ and the orally available K_Ca_3.1 blocker ICA-17043 has been administered to human subjects in phase 2 and 3 trials of sickle cell disease with minor side effects.^40^

In summary, human fibrocytes express the K^+^ channel K_Ca_3.1, which is a key regulator of *in vitro* fibrocyte migration. K_Ca_3.1 blockade may therefore attenuate tissue fibrosis and remodeling in diseases in which fibrocytes play a role, such as IPF and asthma. The availability of a well-tolerated, orally bioavailable K_Ca_3.1 blocker means that this can be tested readily in human clinical trials.

**Key messages**

• Fibrocytes play a key role in tissue remodeling and fibrosis in patients with diseases such as asthma and pulmonary fibrosis. Ion channels are interesting therapeutic targets for the modulation of cell function.
• This is the first investigation of human fibrocyte ion channel expression and demonstrates the presence of functional K_Ca_3.1 K^+^ channels. Activity of these channels is required for fibrocyte migration.
• Inhibition of K_Ca_3.1 ion channels might reduce airway and parenchymal fibrosis in patients with asthma and pulmonary fibrosis, respectively. The availability of an orally bioavailable K_Ca_3.1 blocker means that this can be tested readily in human clinical trials.

## Methods

### Subjects

Healthy control subjects and patients with asthma were recruited from respiratory clinics and hospital staff and by means of local advertising. Asthma was defined by using standard criteria, as described previously.^E1^ Healthy subjects had no history of respiratory disease. All subjects were nonsmokers with a past smoking history of less than 10 pack-years and were free from exacerbations for at least 6 weeks. The Leicestershire Research Ethics Committee approved the study (reference no. 4977). All patients provided written informed consent.

### Cell isolation and culture

Fibrocytes were isolated from peripheral blood and cultured in fibronectin-coated T25 tissue-culture flasks, as described previously.^E2^ After 48 hours, nonadherent cells were removed. After 7 to 14 days, adherent cells were washed with Hanks’ balanced salt solution and harvested with Accutase (eBioscience, Wembley, United Kingdom). Cell counts and viability of the initial isolated PBMC fraction and the final adherent cells were determined by using trypan blue stain. Viability was consistently greater than 95%. In parallel, PBMC preparations were seeded onto fibronectin-coated 8-well chamber slides (2 × 10^5^ cells per well) and cultured as above. Fibrocyte purity and differentiation were assessed by means of flow cytometry and immunofluorescent staining for CD34, αSMA, and collagen I, as described previously.^E2^ Fibrocyte purity was routinely greater than 95%, with morphologically distinct T cells accounting for the contaminants.

### Quantitative real-time RT-PCR

Quantitative real time RT-PCR was performed as described previously.^E3^ Briefly, the following primers for K_Ca_3.1 were used (forward primer, 5′-GGACATCTCCAAGATGCACA-3′; reverse primer, 5′-AGGAGTGGCAGAGACGATGT-3′). The internal normalizer gene used was β-actin (forward primer, 5′-TTCAACTCCATCATGAAGTGTGACGTG-3′; reverse primer, 5′-CTAAGTCATAGTCCGCCTAGAAGCATT-3′). All K_Ca_3.1 expression data were normalized to β-actin and corrected by using the reference dye (ROX). Briefly, PCR was performed with a single-tube Full Velocity SYBR green kit (Stratagene, Amsterdam, The Netherlands). Only experiments in which a distinct single peak was observed with a melting temperature different than that of the no-template control were used. The level of expression between donors was calculated by the cycle threshold of the gene of interest/cycle threshold of the internal normalizing gene.

### Analysis of K_Ca_3.1 protein expression

K_Ca_3.1 protein expression was analyzed by means of Western blotting, as previously described.^E4^ Soluble protein from equivalent numbers of cells was resolved by using 12% SDS-PAGE and then transferred to a nitrocellulose membrane. Membranes were blocked and incubated with validated primary rabbit anti-human K_Ca_3.1 antibodies (M20; a gift from Dr M. Chen, GlaxoSmithKline)^E5^ and P4997 (Sigma-Aldrich). Protein bands were identified by means of horseradish peroxidase–conjugated secondary antibody (DAKO, Cambridge, United Kingdom) and ECL reagent (Amersham, Little Chalfont, United Kingdom).

K_Ca_3.1 expression was also examined by means of immunofluorescence with the same M20 and P4997 antibodies, with methods as described previously.^E4^

### Patch-clamp electrophysiology

The whole-cell variant of the patch-clamp technique was used.^E6,E7^ Patch pipettes were made from borosilicate fiber–containing glass (Clark Electromedical Instruments, Reading, United Kingdom), and their tips were heat polished, typically resulting in resistances of 4 to 6 MΩ. The standard pipette solution contained 140 mmol/L KCl, 2 mmol/L MgCl_2_, 10 mmol/L N-2-hydroxyethylpiperazine-N-2-ethanesulfonic acid (HEPES), 2 mmol/L Na^+^-ATP, and 0.1 mmol/L guanosine triphosphate (pH 7.3). The standard external solution contained 140 mmol/L NaCl, 5 mmol/L KCl, 2 mmol/L CaCl_2_, 1 mmol/L MgCl_2_, and 10 mmol/L HEPES (pH 7.3). For recording, fibrocytes were placed in 35-mm dishes containing standard external solution. Whole-cell currents were recorded with an Axoclamp 200A amplifier (Axon Instruments, Foster City, Calif), and currents were evoked by applying voltage commands to a range of potentials in 10-mV steps from a holding potential of −20 mV. The currents were digitized (sampled at a frequency of 10 kHz), stored on computer, and subsequently analyzed with pClamp software (Axon Instruments). Capacitance transients were minimized by using the capacitance neutralization circuits on the amplifier. Correction for series resistance was not routinely applied. Experiments were performed at 27°C, and the temperature was controlled with a Peltier device. Experiments were performed with a perfusion system (Automate Scientific, San Francisco, Calif) to allow solution changes, although drugs were added directly to the recording chamber.

### Fibrocyte migration

First, we used a 24-well transwell migration assay to measure the migration of detached differentiated fibrocytes that had been in culture for 1 week after isolation. The transwell inserts were coated with 40 μg/mL fibronectin for 1 hour at 37°C. Chemoattractant was added to the bottom compartment of each well, with the exception of the negative controls. For the chemoattractant, we used 425 μL of conditioned medium from ASM cultures that had been activated for 24 hours with 10 ng/mL TNF-α. Chemokines present in this medium include the fibrocyte chemoattractant CXCL12.^E8^ The ASM supernatant was supplemented with 5% serum. Fibrocyte cultures were thoroughly washed several times with fresh medium to remove nonadherent cells. Cultures were then trypsinized, and 1 × 10^5^ fibrocytes were added to the top chamber of each well in duplicate. Cells were incubated in the presence of chemoattractant for 4 hours, at which point the cells present in the lower well were recovered by means of pipetting, transferred to a 96-well V-bottom plate, centrifuged at 250*g* for 5 minutes, and then resuspended in Kimura stain for counting with a hemocytometer by a blinded observer. Pilot studies demonstrated that fibrocytes were readily distinguishable from sparse (<5% of migrating cells) T cells based on morphology, did not adhere to the underside of the transwell membrane, and did not remain adherent to the lower chamber in the short term after pipetting. Where required, the specific K_Ca_3.1 blockers TRAM-34 (a gift from Professor H. Wulff, University of California, Davis, Calif)^E9^ and ICA-17043 (a gift from Icagen, Inc)^E10^ were added to the top chamber before migration.

In a further set of migration experiments, freshly isolated PBMCs containing the fibrocyte population were allowed to migrate through a transwell, as described above (1 × 10^7^ cells/top chamber), with the established fibrocyte chemoattractant CXCL12^E11^ placed in the lower chamber. Where required, the K_Ca_3.1 blocker TRAM-34 was added to the top chamber before migration. After 4 hours, the transwell was discarded, and the migrated cells were cultured for 24 hours, washed to remove nonadherent cells, and then cultured for a further 6 days. The cells were then washed 3 times to remove nonadherent cells, and the remaining adherent cells were fixed in 4% paraformaldehyde, stained for collagen I, and then counted by a blinded observer.

A second migration assay was used to investigate the migration of adherent elongated fibrocytes, as described previously.^E2^ ASM cells (2.5 × 10^5^ cells per well) were seeded onto fibronectin-coated (1 μg/mL) 8-rectangular-well plates and allowed to adhere overnight before removal by scraping of half the ASM below a predrawn line on the well underside. Fibrocytes (4-8 × 10^4^ cells per well) were then added for 72 hours before pretreatment with the specific K_Ca_3.1 blockers TRAM-34 and ICA-17043 for 1 hour before migration tracking began. Photographs of the fibrocytes were taken along a predrawn line 5 mm below the edge of the multicell layer or equivalent position in control wells at 1.5-hour intervals over a 4.5-hour time course. The migration of individual fibrocytes was tracked manually, and both the distance of migration and the percentage of cells migrating were measured by a blinded observer.

### Fibrocyte growth and maturation

Fibrocytes were incubated with TRAM-34 at a dose range of 20 to 2000 nmol/L in their normal growth medium, either from days 0 to 7 after isolation or from days 7 to 14 after isolation. Cells were then harvested and counted with a hemocytometer. In addition, the MTS proliferation assay was performed. Fibrocytes were cultured at a density of 2 × 10^3^ cells per well in flat-bottomed 96-well plates either for 24 hours or 7 days and then serum starved for 24 hours. Fibrocytes were then incubated with or without TRAM-34 (dose range, 20-2000 nmol/L) in their normal growth medium for a further 7 days. Next, 20 μL of CellTiter 96 Aqueous One Solution Reagent (Promega, Southampton, United Kingdom) was added to each well. After 4 hours in culture, the cell viability was determined by measuring the absorbance at 490 nm with a Wallac Victor^E2^ 1420 multilabel counter (PerkinElmer, Cambridge, United Kingdom).

Parallel chamber slides were examined to examine fibrocyte morphology and differentiation markers, as described previously.^E2^

### Quantification of cell death by means of flow cytometry

After isolation, fibrocytes were incubated with TRAM-34 at a dose range of 20 to 2000 nmol/L in their normal growth medium, either from days 0 to 7 after isolation or from days 7 to 14. Fibrocytes were harvested by means of trypsinization to determine the percentage of apoptotic or necrotic cells. Next, these cells were suspended in Annexin-binding buffer containing fluorescein isothiocyanate–conjugated Annexin V (5 μL/300 μL of binding buffer; BD PharMingen, San Jose, Calif), followed by counterstaining with or without propidium iodide (0.5 μg/mL), before processing on the BD FACSCanto flow cytometer (BD Biosciences, Oxford, United Kingdom). The intensity of staining was determined for 10,000 cells in each assay. Thereafter, the samples were analyzed by means of flow cytometry for the presence of early apoptotic (only Annexin V positive) and late apoptotic (double positive for Annexin V and propidium iodide) cells.

### Statistics

Experiments were performed in duplicate, and a mean value was derived for each condition. For all assays, across-group differences were analyzed by means of ANOVA, and inhibition of migration was analyzed by using the paired *t* test. Patch-clamp data were analyzed by using paired or unpaired *t* tests, as appropriate.

## Results

### Human fibrocytes express robust K_Ca_3.1 ion currents

To elicit K_Ca_3.1 currents, we used the K_Ca_3.1 opener 1-EBIO (Tocris, Avonmouth, United Kingdom), as described previously in human lung mast cells^E12,13^ and ASM cells.^E3^ This compound opens K_Ca_3.1 with a half-maximal value of about 30 μmol/L for heterologously expressed K_Ca_3.1, with a maximal effect at 300 μmol/L.^E14^

Addition of 1-EBIO (100 μmol/L) elicited a typical K_Ca_3.1 current in 46 of 47 cells tested (7 healthy and 4 asthmatic donors). Whole-cell current at +40 mV increased from a mean ± SEM of 122 ± 25 pA before 1-EBIO to 795 ± 104 pA after 1-EBIO (*P* < .0001). This was accompanied by a negative shift in reversal potential from −29.8 ± 3.0 mV to −61.8 ± 1.6 mV (*P* < .0001). 1-EBIO induced a greater current in fibrocytes from asthmatic patients (1-EBIO–dependent current at +40 mV = 969 ± 216 pA [n = 17 cells]) compared with fibrocytes from healthy subjects (1-EBIO–induced current = 506 ± 94 pA [n = 30 cells], *P* = .029), but there was no difference in reversal potential (*P* = .352).

The 1-EBIO–induced current demonstrated the classical electrophysiological features of K_Ca_3.1 in that it appeared immediately as voltage steps were applied, did not decay during a 100-ms pulse, and demonstrated inward rectification from membrane potentials positive to about +40 mV. Furthermore, the current was completely blocked by the selective K_Ca_3.1 blockers TRAM-34^E9^ and ICA-17043^E10^ at concentrations of 200 and 100 nmol/L, respectively. Thus 1-EBIO–induced current at +40 mV was reduced from 984 ± 226 pA after 1-EBIO to 171 ± 64 pA after 200 nmol/L TRAM-34 (n = 18 cells, *P* = .0005), with a positive shift in reversal potential from −65.4 ± 1.7 mV after 1-EBIO to −12.7 ± 2.3 mV after TRAM-34 (*P* < .0001). With ICA-17043, the 1-EBIO−induced current at +40 mV reduced from 774 ± 203 pA to 128 ± 52 pA after 100 nmol/L ICA-17043 (n = 8 cells, *P* = .0072), with a shift in reversal potential from −59.3 ± 4.5 mV after 1-EBIO to −12.5 ± 6.7 mV after ICA-17043 (*P* = .0016). The reversal potentials after addition of TRAM-34 and ICA-17043 (in the presence of 1-EBIO) were significantly more positive (*P* = .0018 and *P* = .0016, respectively) than the respective baseline values, indicating the presence of open K_Ca_3.1 channels at rest. In summary, human fibrocytes express robust K_Ca_3.1 currents.

In keeping with previous findings in human lung mast cells, in which we studied CXCL10,^E15^ K_Ca_3.1 was not opened in fibrocytes by the chemokine CXCL12. This indicates that the gating of K_Ca_3.1 during fibrocyte migration is downstream of the chemoattractant stimulus, most probably related to adhesive signals required for the migratory process.
